# Loss of Photoreceptors Results in Upregulation of Synaptic Proteins in Bipolar Cells and Amacrine Cells

**DOI:** 10.1371/journal.pone.0090250

**Published:** 2014-03-04

**Authors:** Sushma Dagar, Saumya Nagar, Manvi Goel, Pitchaiah Cherukuri, Narender K. Dhingra

**Affiliations:** National Brain Research Centre, Manesar (Gurgaon) Haryana, India; Tokai University, Japan

## Abstract

Deafferentation is known to cause significant changes in the postsynaptic neurons in the central nervous system. Loss of photoreceptors, for instance, results in remarkable morphological and physiological changes in bipolar cells and horizontal cells. Retinal ganglion cells (RGCs), which send visual information to the brain, are relatively preserved, but show aberrant firing patterns, including spontaneous bursts of spikes in the absence of photoreceptors. To understand how loss of photoreceptors affects the circuitry presynaptic to the ganglion cells, we measured specific synaptic proteins in two mouse models of retinal degeneration. We found that despite the nearly total loss of photoreceptors, the synaptophysin protein and mRNA levels in retina were largely unaltered. Interestingly, the levels of synaptophysin in the inner plexiform layer (IPL) were higher, implying that photoreceptor loss results in increased synaptophysin in bipolar and/or amacrine cells. The levels of SV2B, a synaptic protein expressed by photoreceptors and bipolar cells, were reduced in whole retina, but increased in the IPL of rd1 mouse. Similarly, the levels of syntaxin-I and synapsin-I, synaptic proteins expressed selectively by amacrine cells, were higher after loss of photoreceptors. The upregulation of syntaxin-I was evident as early as one day after the onset of photoreceptor loss, suggesting that it did not require any massive or structural remodeling, and therefore is possibly reversible. Together, these data show that loss of photoreceptors results in increased synaptic protein levels in bipolar and amacrine cells. Combined with previous reports of increased excitatory and inhibitory synaptic currents in RGCs, these results provide clues to understand the mechanism underlying the aberrant spiking in RGCs.

## Introduction

Loss of photoreceptors, as in retinal degenerative diseases, leads to remarkable morphological and physiological remodeling in the second-order neurons, namely, bipolar cells and horizontal cells [1]–[9]. There is growing evidence that even the inner retinal neurons and their synaptic interactions in the inner plexiform layer (IPL) exhibit significant alterations early on. For example, bipolar cell axon terminals show early signs of degeneration [10]–[11]. Similarly, amacrine cells have been shown in various animal models to exhibit dendritic sprouting, abnormal morphology, aberrant glutamate response and altered contribution to electroretinogram (ERG) [11]–[15]. Retinal ganglion cells (RGCs), although they maintain their intrinsic physiological properties, show altered receptive field properties and aberrant spontaneous firing [16]–[17]. For example, following photoreceptor loss many RGCs exhibit spontaneous bursts of spikes [18]–[19] which have been reported to originate in the bipolar cell – amacrine cell network [16], [20]–[23].

To further understand how loss of photoreceptors affects the synaptic machinery in inner retina, we measured levels of several synaptic proteins and their mRNAs in two animal models of retinal degeneration. These proteins, namely, synaptophysin, SV2B, syntaxin-I and synapsin-I, are involved in several important synaptic functions, such as vesicle movement, docking and fusion that eventually lead to neurotransmitter release [24]–[28]. These specific proteins were chosen because they are differentially expressed in retinal neurons, and therefore are likely to provide insights about synaptic changes in specific inner retinal neurons. Synaptophysin, a synaptic vesicle protein is expressed in all retinal vesicular synapses [29]–[31]. In contrast, SV2B is expressed selectively in ribbon synapses of photoreceptors and bipolar cells [32]–[33] while synapsin-I and syntaxin-I are expressed selectively in conventional synapses of amacrine cells [31], [34]–[36]. Some of these proteins have been studied in various animal models of retinal degeneration [8], [37]–[39]. However, these studies were either qualitative or quasi-quantitative (see Discussion).

It is important to study the synaptic remodeling in inner retina after photoreceptor loss, because it would not only reveal the pathophysiology of retinal degeneration, but also help understand how inner retinal circuitry functions normally. Furthermore, this has implications for some of the experimental therapeutic strategies for retinal degeneration. For example, the cell-based or bionic approaches to restore vision after photoreceptor loss require morphological and physiological integrity of the inner retinal circuitry [17], [40]–[43]. Understanding the synaptic changes in inner retina would help determine if these approaches require additional therapeutic targets, for example, to normalize the spontaneous bursts of spikes observed in RGCs after photoreceptor loss.

## Materials and Methods

### Ethics Statement

All experiments were approved by the Institutional Animal Ethics Committee of the National Brain Research Centre. All efforts were made to minimize the number of animals used and their suffering.

### Animals, drug administration and tissue preparation

Wild-type (C57BL/6J) and rd1 (PDE6b^rd1^; CBA/J) mice were obtained from Jackson Laboratory (Bar Harbor, USA), and bred locally at the animal facility of the National Brain Research Centre, India. Animals were maintained on a 12-hour light/dark cycle.

Some adult (2–3 months) wild-type male mice were injected with N-methyl-N-nitrosourea (MNU) (i/p; 65 mg/kg in physiological saline containing 0.05% acetic acid; Sigma-Aldrich, St. Louis, USA or Chemservice, West Chester, USA) [8], [44]–[45]. Control mice were injected with physiological saline containing 0.05% acetic acid. These animals were enucleated after cervical dislocation at 1, 2, 4, 7, 14 or 28 days after the injection. Similarly, wild-type and rd1 mice (both male and female) were sacrificed at postnatal day 7, 14, 21, 28 or 2–3 months. Retinas were then processed for protein extraction, cryosectioning or RNA isolation.

For protein extraction, the eyes were hemisected in ice-cold phosphate-buffered saline (PBS) containing 10 mM ethylenediaminetetraacetic acid (EDTA; pH 8.0) and retinas were collected in protein lysis buffer (75 µl per retina) containing 50 mM Tris (pH 7.5), 150 mM sodium chloride, 1 mM EDTA, 50 mM sodium fluoride, 1 mM sodium orthovandate, 2% sodium dodecyl sulfate (SDS) and a cocktail of protease inhibitors (Complete Protease Inhibitor cocktail; Roche Applied Science, Penzberg, Germany) on ice, as described previously [8]. Retinas were then sonicated and centrifuged at 12000 rpm for 30 minutes. Supernatant was collected and protein levels were estimated using bicinchoninic acid method (BCA kit, Sigma-Aldrich).

For cryosectioning, an eyeball was given a small incision, pre-fixed in 4% paraformaldehyde for 10 min at 4°C, hemisected and post-fixed in 4% paraformaldehyde for 45 min at 4°C. The eyecup was then kept in 30% sucrose at 4°C for cryopreservation. The eyecup was embedded in Optimal Cutting Temperature compound for sectioning. Sections of 10 µm thickness were cut using a cryostat (model CM3050S, Leica, Wetzlar, Germany). Sections from the mid-peripheral retina were used for immunohistochemistry.

For RNA isolation, retinas were removed, collected in ice cold Trizol (Invitrogen, Carlsbad, USA) and homogenized using a rotor-stator homogenizer (IKA, Staufen, Germany). Total RNA was isolated using phenol-chloroform extraction method as described previously [46].

### Primary antibody characterization

**Figure 1:**
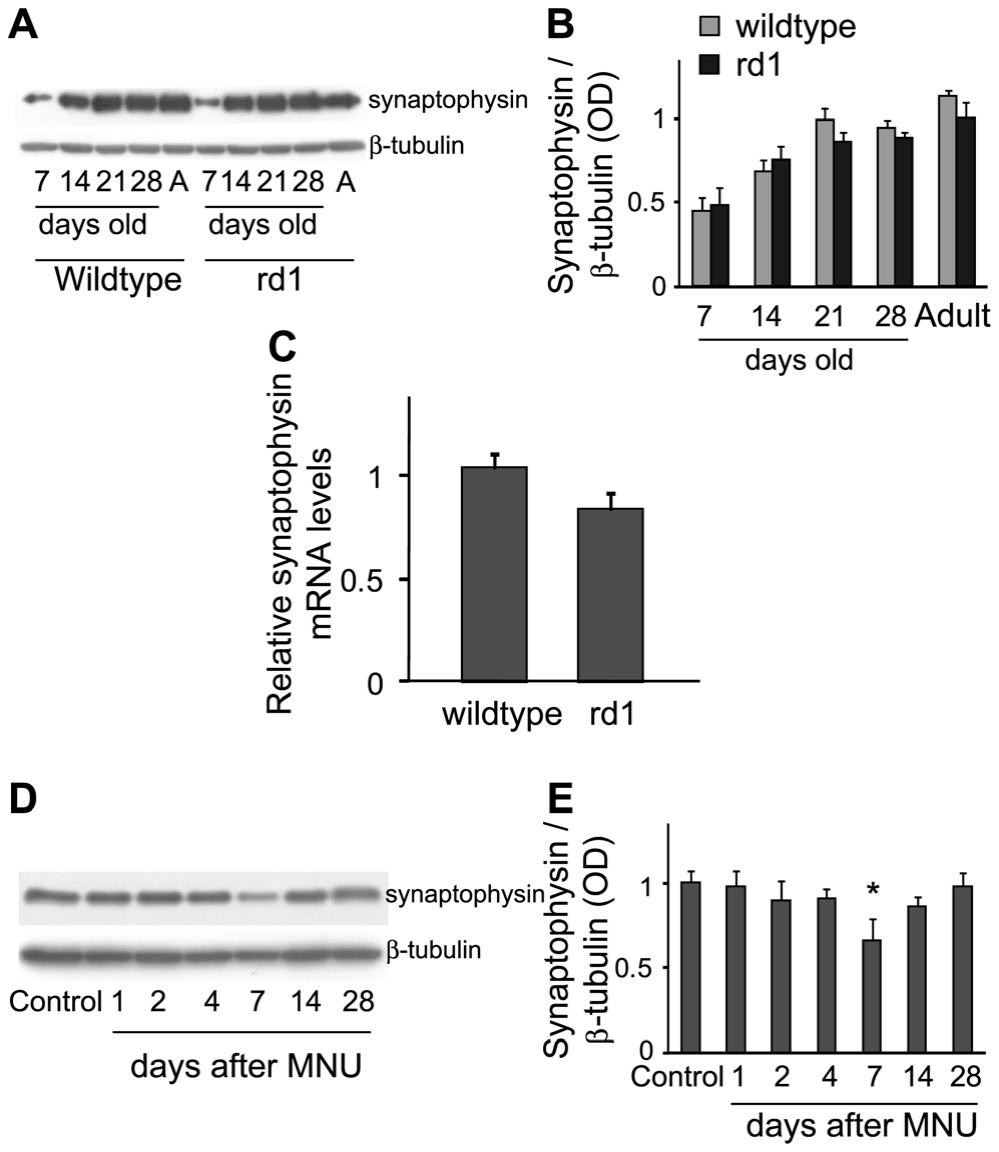
Retinal levels of synaptophysin protein and mRNA were largely unaltered following photoreceptor loss. A) Representative blots of synaptophysin and β-tubulin in whole retinas of wild-type and rd1 mice at different developmental stages (“A” is for “Adult”). B) Ratio of synaptophysin to β-tubulin for several animals (Mean ± SE). The ratio in rd1 mouse was not statistically different from that in wild-type mouse at any stage (n = 6, 8, 9, 9 and 7 for 7, 14, 21, 28 days old and adult animals respectively). C) Synaptophysin mRNA levels normalized to 18S rRNA (Mean ± SE) were also similar in adult rd1 and wild-type mice (n = 6). D) Representative blots of synaptophysin and β-tubulin in whole retinas of sham-injected control and at various days after MNU injection. E) Ratio of synaptophysin to β-tubulin for several animals (Mean ± SE). Similar to rd1 mouse, the levels of synaptophysin in MNU-injected animals were not significantly different from the control for up to at least 28 days after the injection, except at 7 days (p>0.1, except for PID-7 where p = 0.044; n = 6 for all stages). *p<0.05

**Figure 2:**
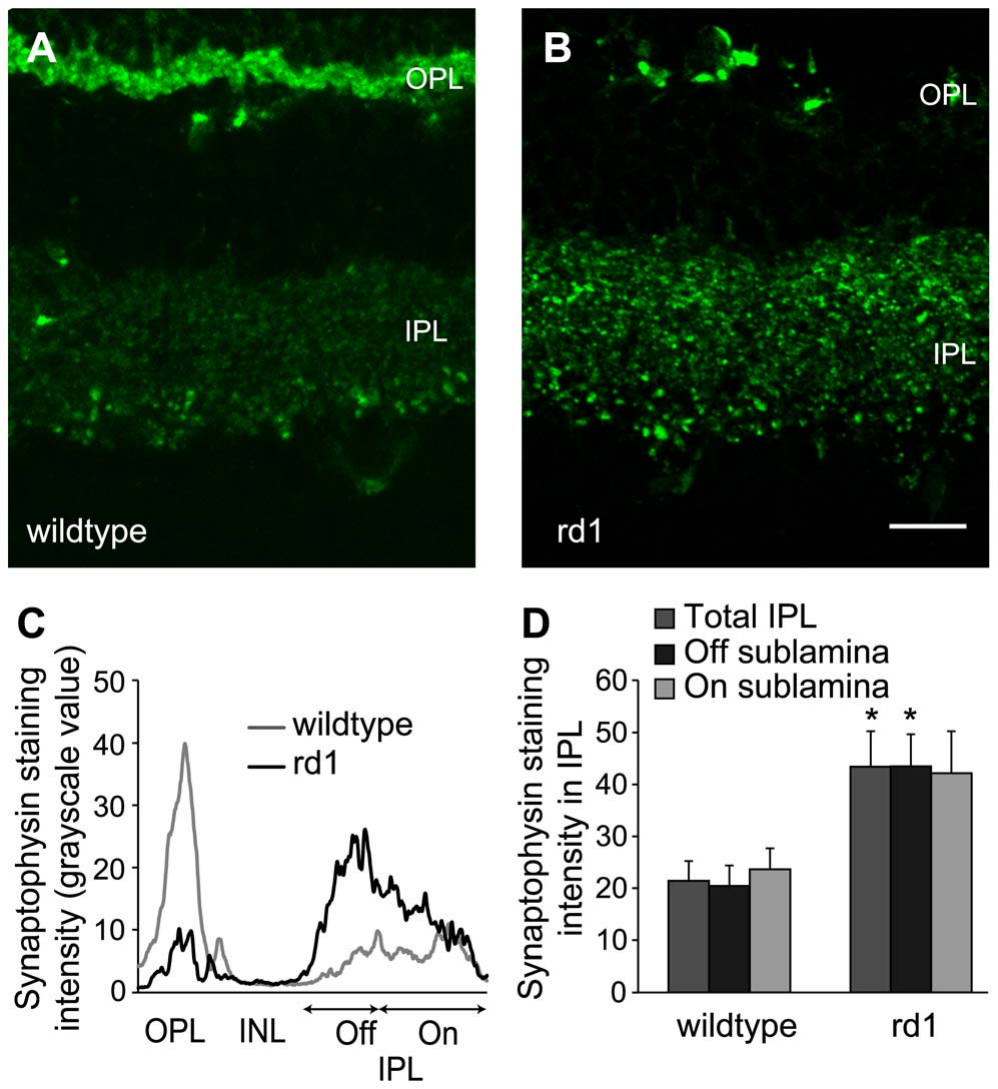
Synaptophysin in the IPL of rd1 mouse was upregulated. A, B) Representative images of vertical sections of adult wild-type (A) and rd1 (B) mouse retinas immunostained for synaptophysin. Scale bar: 50 µm. C) Representative profile of staining intensity through retinal depth covering both plexiform layers of the images shown in A and B. After background subtraction, a line moving vertically from top to bottom across the images measured the signal intensity using Plot Profile function in ImageJ, which is shown here from left to right. Synaptophysin staining is nearly absent in OPL of rd1 mouse retina, whereas that in IPL is higher than in wild-type, particularly in the Off sublamina. D) Levels of synaptophysin in IPL (mean ± SE), measured with quantitative immunohistochemistry were significantly higher in adult rd1 mouse retina than in wild-type (p = 0.018; n = 6). Within IPL, the levels were significantly higher in Off sublamina (p = 0.01), but not in On sublamina (p = 0.068). The data shown here are essentially the area under the curve for On (60% of IPL) and Off (40%) sublaminas shown in 2C. *p<0.05.

The primary antibodies used here are described in Table 1. Synaptophysin is a synaptic vesicle protein expressed by all chemical synapses in mammalian retina [30]. The synaptophysin antibody used here detects a single band at 38 kDa (manufacturer's data; see Figs. 1A, 1C), which was used for the densitometric analysis. It produces bright punctate labeling in both outer plexiform layer (OPL) and IPL in mouse retina (see Figs. 2A, 2B).

**Table 1:** List of antibodies used.

Antibody	Immunogen	Manufacturer, Catalog no	Species, type	dilution
Synaptophysin	A synaptosome preparation from rat retina	Sigma-Aldrich (St. Louis, USA) cat. # S5768	Mouse monoclonal	1:5000 (Immunohistochemistry and Western blotting)
SV2B	Synthetic peptide DDYRYRDNYEGYAPND (aa 2–17 in rat) coupled to key-hole limpet hemocyanin via an added N-terminal cysteine residue	Synaptic systems (Goettingen, Germany) cat.#119111	Mouse Monoclonal	1:4000 (Immunohistochemistry); 1:12000 (Western blotting)
Syntaxin-I	GST fusion protein corresponding to a cytoplasmic part of rat Syntaxin IA (residue 1–265) (Accession number P32851)	Chemicon (Billerica, USA) cat. # AB5820-50UL	Rabbit polyclonal	1:12000 (Western blotting)
Syntaxin	Synaptic vesicle fractions that were immuo-precipitated from human brain homogenates using anti-human synaptophysin monoclonal antibodies	Stressgen (Victoria, Canada) cat.# VAM-SV013	Mouse monoclonal	1:12000 (Western blotting)
Synapsin-I	Synapsin I purified from bovine brain	Sigma-Aldrich cat.# S193	Rabbit polyclonal	1:10000(Western blotting)
β-tubulin	Tubulin from rat brain	Sigma-Aldrich cat.# T4026	Mouse Monoclonal	1:12000 (Western blotting)

**Figure 3:**
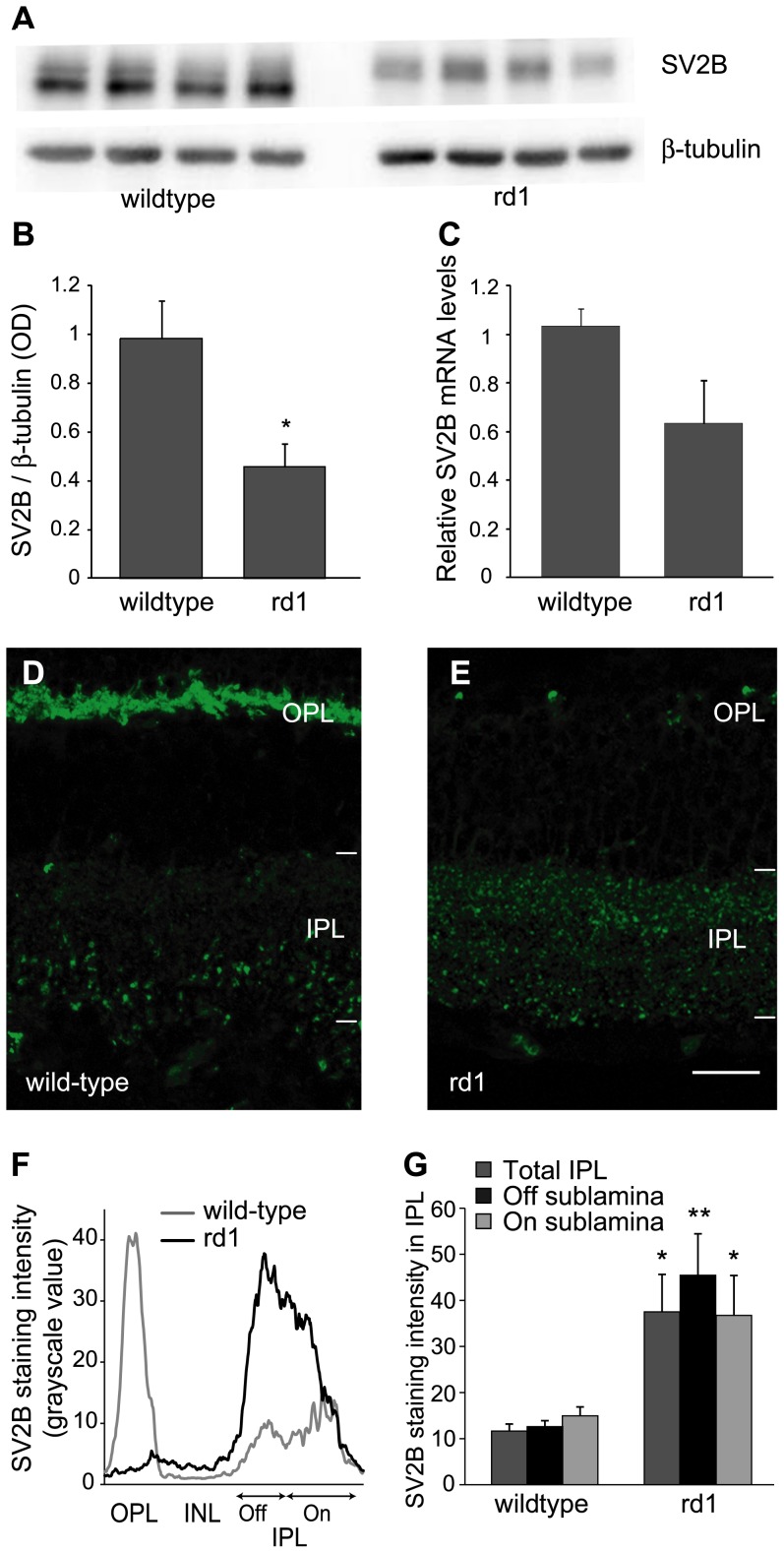
Ribbon-specific protein SV2B was downregulated in retina, but upregulated in IPL of rd1 mouse. A) Representative blots of SV2B and β-tubulin in adult wild-type and rd1 mouse retinas. Each band is from a different animal. B) Ratio of SV2B to β-tubulin (mean ± SE) in adult rd1 mouse retina was significantly lower than in wild-type mouse (p = 0.01; n = 5). C) Relative mRNA levels (mean ± SE) of SV2B in adult rd1 mouse were approximately 40% lower than in wild-type, although the difference was not statistically significant (p = 0.062; n = 6). D, E) Representative images of retinal sections of adult wild-type (D) and rd1 (E) mouse retinas immunostained for SV2B. Scale bar: 50 µm. A) Intensity profile of SV2B through retinal depth of the images shown in D and E. Similar to synaptophysin (Figs. 2A, 2B, 2C), SV2B is nearly absent in OPL of rd1 mouse retina, whereas that in IPL is higher than in wild-type, particularly in the Off sublamina. B) Levels of SV2B (mean ± SE), measured with quantitative immunohistochemistry were more than twofold higher in IPL of adult rd1 mouse retina than in wild-type (p = 0.014; n = 5). The SV2B levels were higher in both On and Off sublaminas of IPL, although the increase was more pronounced in the Off sublamina (On: p = 0.031; Off: p = 0.005). *p<0.05; **p<0.01.

SV-2B antibody used here detects two indistinct or a thick smeary band near 75 kDa in retina (manufacturer's data/emails, Fig 3A) and is known to label ribbon synapses in the mouse retina. The reason for two indistinct bands is not clear, but could be related to differential glycosylation of this protein in retina. A characteristic punctate staining in the OPL and IPL [33] (see Figs. 3D, 3E) confirmed the antibody specificity.

**Figure 4:**
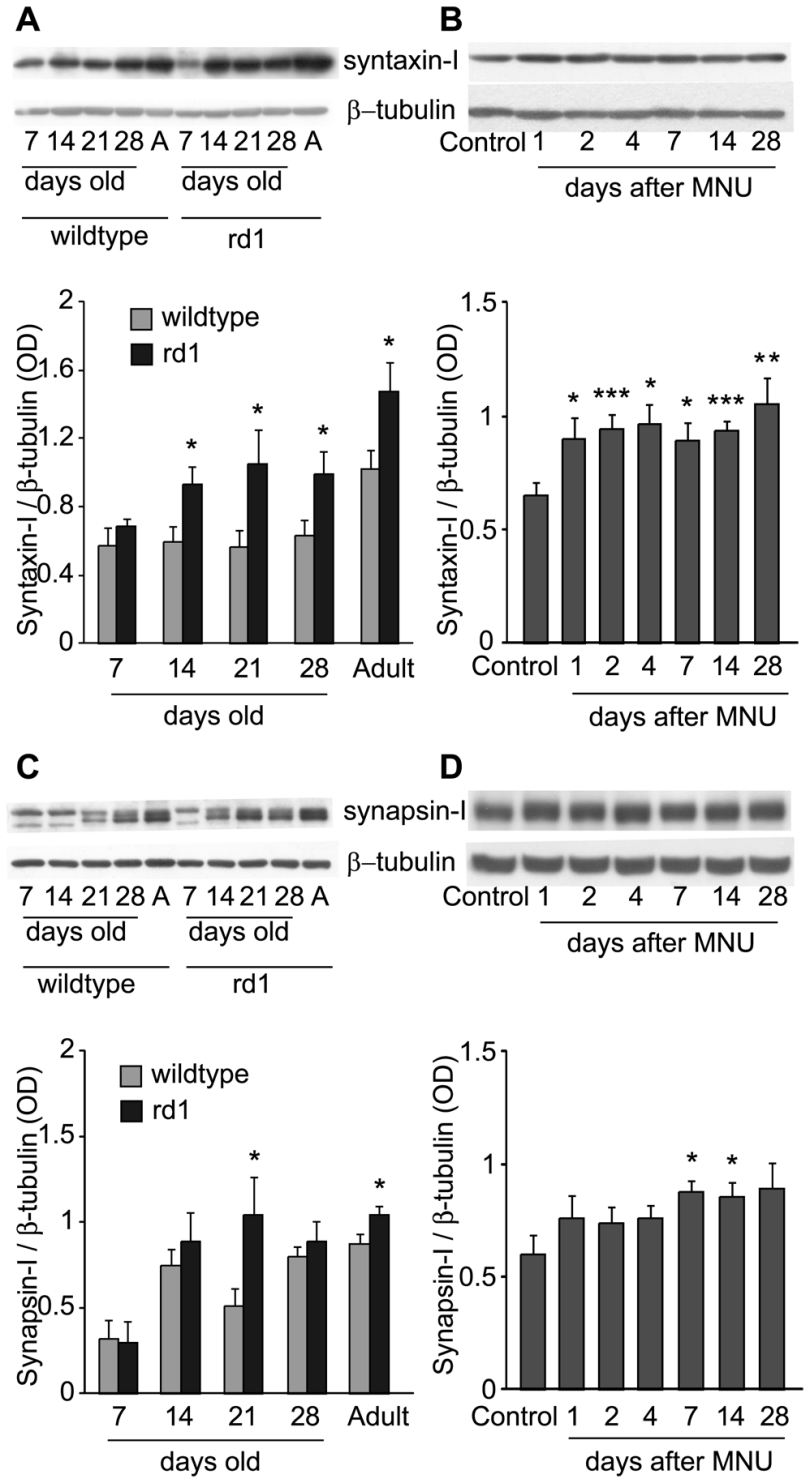
Amacrine cell-specific synaptic proteins were upregulated following photoreceptor loss. A) *Top*: Representative blots of syntaxin-I and β-tubulin in retinas of wild-type and rd1 mice at different developmental stages (“A” is for “Adult”). *Bottom*: Ratio of syntaxin-I to β-tubulin for several animals (Mean ± SE). Syntaxin-I was upregulated in rd1 mouse retina from P-14 onwards (n = 7, 5, 7, 10, 14 for 7, 14, 21, 28 days old and Adult animals respectively). B) *Top*: Representative blots of syntaxin-I and β-tubulin in retinas of sham-injected control and at various days after MNU injection. *Bottom*: Ratio of syntaxin-I to β-tubulin for several animals (Mean ± SE). Similar to rd1 mouse, the levels of syntaxin-I were significantly higher in MNU-injected mice than in sham-injected controls. The upregulation was evident as early as one day after the injection (n = 10 for control; 9 for PID-1; 11 for PID-2, PID-4, PID-7 and PID-14; 10 for PID-28). C) *Top*: Representative blots of synapsin-I and β- tubulin in retinas of wild-type and rd1 mice at different developmental stages. *Bottom*: Ratio of synapsin-I to β-tubulin for several animals (Mean ± SE). Synapsin-I was upregulated in rd1 mouse from P-21 onwards till adult stage, although it was not statistically significant at P-28 (n = 4, 6, 8, 9 and 12 for 7, 14, 21, 28 days old and adult animals respectively). D) *Top*: Representative blots of synapsin-I and β-tubulin in retinas of sham-injected control and at various days after MNU injection. *Bottom*: Ratio of synapsin-I to β-tubulin for several animals (Mean ± SE). Similar to rd1 mouse, the levels of synapsin-I were significantly higher in MNU-injected mice than in sham-injected controls. The upregulation was evident from 7 days onwards after the injection (n = 7 for control; 6 for PID-1; 8 for PID-2, PID-4, PID-7 and PID-28; 9 for PID-14). *p<0.05; **p<0.01; ***p<0.005.

Syntaxin-I is a membrane protein which forms soluble *N*-ethylmaleimide-sensitive factor attachment protein (SNAP) receptor (SNARE) complex that mediates vesicular trafficking to the plasma membrane and is expressed by the conventional synapses in IPL and the amacrine cell bodies in inner nuclear layer (INL) [36]. Immunoblot shows a single band at ∼37 kDa which was considered for densitometric analysis (manufacturer's data; see Figs. 4A, 4B). For some experiments, we used a mouse monoclonal syntaxin antibody (Stressgen, Victoria, Canada; Cat # VAM-SV013). A double immunolabeling of retinal sections for this and syntaxin-I antibody revealed complete co-localization, implying that they recognized the same epitope (data not shown). Furthermore, the syntaxin-I peptide (Chemicon, Cat # AB5820) completely blocked the syntaxin labeling, further confirming the specificity of this antibody.

Synapsin-I is a synaptic vesicle associated protein which is involved in clustering the synaptic vesicles and is expressed in the conventional synapses of amacrine cells in mouse retina [34], [47]. The synapsin-I antibody used here labeled IPL (not shown) and detects a double band at ∼80 kDa (synapsin-Ia) and 77 kDa (synapsin-Ib) (manufacturer's data; see Fig.4C). Both the bands were considered for the quantification.

β-tubulin antibody was used as loading control for western blotting. This antibody detects a single band at 55 KDa which was considered for the quantification.

### Immunoblotting

Immunoblotting was carried out as described previously [8], [46]. Briefly, a 10 µg of protein sample was electrophoresed in SDS-polyacrylamide gel and electroblotted to activated polyvinylidene difluoride (PVDF) membrane (MDI Membrane Technologies, Ambala, India). The blots were incubated for one hour at room temperature in blocking solution (5% bovine serum albumin (BSA) in Tris-buffered saline (TBS) containing 0.1% Tween-20, pH 7.4), followed by overnight incubation at 4°C in a primary antibody (Table 1) in the blocking solution. Primary antibodies were detected with appropriate horseradish peroxidase-conjugated secondary antibodies (1∶4000, Vector Laboratories, Burlingame, USA). The blots were washed 3×10-min in TBS and tween-20 (TBST), after primary and secondary antibody incubation. The signals were detected using enhanced chemiluminescence (ECL; Millipore, Billerica, USA) method on photographic films (Amersham Hyperfilm ECL, GE Healthcare, Little Chalfont, UK) or with GelDoc (Universal Hood II, Bio-Rad Hercules, USA). Densitometric analysis was performed with ImageJ software (National Institutes of Health, USA). The grayscale image was “inverted” and the background was uniformly subtracted wherever necessary. The signal of each protein was normalized with the loading control (β-tubulin), and the data were expressed as integrated optical density (OD).

### Quantitative Immunohistochemistry

Quantitative immunohistochemistry was carried out based on previous reports [48]–[54]. Retinal sections were washed 2×5-min in PBS, and incubated in blocking solution (3% normal donkey serum, 3% BSA, 0.3% Triton X-100 in PBS) in a dark humidified chamber for one hour at room temperature. Sections were then incubated with the primary antibody (Table 1) overnight at 4°C. The optimal concentration of a primary antibody was determined by using a range of antibody concentrations, plotting the staining intensity against the concentration, and selecting from linear part of the curve the concentration that produced approximately half-maximum intensity. Primary antibody incubation was followed by incubation in AlexaFluor488-conjugated anti-mouse secondary antibodies (Invitrogen, Carlsbad, US) for one hour at room temperature. The secondary antibodies were diluted in the blocking solution (1∶500). Sections were washed 3×5-min in PBS after primary and secondary incubations. Sections were then mounted with Vectashield containing 4′,6-diamidino-2-phenylindole (DAPI) (Vector Laboratories). Each pair of control and test samples was processed simultaneously, keeping all the conditions same. Stained sections were imaged with Argon laser in z-axis as 1 µm optical serial sections using a confocal microscope (LSM 510 Meta; Carl Zeiss, Oberkochen, Germany). Images were captured using a 40x oil-immersion lens (NA 1.3), keeping the pinhole size at 1.0 AU. The detector gain and offset values were optimized manually, and kept constant for each pair of control and test samples. To minimize the variability from the antibody solutions penetrating the deeper layers of the sections, only top five (out of 5–7) optical sections were taken and stacked offline using ImageJ. The intensity was calculated by marking the IPL boundary and measuring the mean gray values after uniform background subtraction, using the rolling ball radius as 150 pixels in the 8-bit grayscale images. For each sample, 20–25 images were used for quantification.

### Quantitative Real time PCR (qRT-PCR)

The mRNA was reverse-transcribed using cDNA synthesis kit as per the manufacturer's protocol (Roche Applied Science). Briefly, a 500 ng of cDNA was used as the starting material. The mRNA levels were measured by real-time PCR using Power SYBR Green PCR Master Mix (Applied Biosystems) in accordance with the manufacturer's protocol. Real-time PCR conditions were as follows: 95°C for 10 min (1 cycle), denaturation at 95°C for 20 sec, annealing for 30 sec, and extension at 72°C for 40 sec (40 cycles). Annealing temperature used for synaptophysin, SV2B and synapsin-I was 58°C, and for syntaxin-IA was 60°C. All reactions were carried out in duplicate, and negative controls without template were run simultaneously. The dissociation curves were generated to check for the specificity of primer annealing to the template. Data were analyzed using comparative threshold cycle (δδCt) method (relative quantification). Ct values obtained from real time PCR were normalized with 18S rRNA (internal control). We used the following primers (5 µM):

syntaxin-1A: 5′GAAGACGCAGCACTCCACGCT 3′(forward) and 5′ATGGCAGGGTTCCCACTCTCCAG3′ (reverse), synapsin-I: 5′TGCTGGCGGATCAGCACAAAGT 3′ (forward) and 5′CCGCACACCGACTGGGCAAAATA3′ (reverse), synaptophysin: 5′AGGTGCTGCAGTGGGTCTTTGC 3′ (forward), and 5′ CCCCTTTAACGCAGGAGGGTGC3′ (reverse), 18S rRNA: 5′GAGGGAGCCTGAGAAACGG 3′ (forward) and 5′ GTCGGGAGTGGGTAATTTGC 3′ (reverse), SV2B: 5′ ACCATTTTCAAACAGGTCTGGG 3′ (forward) and 5′ATAGCGGATCATGTCGGGGA 3′ (reverse).

### Statistical analyses

Statistical comparisons between control and test samples were made using unpaired two-tailed *t-test* and p values less than 0.05 were considered significant. The data are expressed as mean±SE.

## Results

### Synaptophysin was unaltered in whole retina, but upregulated in inner retina following photoreceptor loss

Quantitative immunoblot analysis revealed that the synaptophysin levels in the retinas of developing and adult rd1 mice (OD values; mean ± SE; P-7: 0.49±0.1, P-14: 0.75±0.09, P-21: 0.86±0.06, P-28: 0.89±0.03, adult: 1.01±0.1), although slightly reduced, were not significantly different from those in age-matched wild-type mice (0.45±0.08, 0.68±0.07, 0.99±0.07, 0.95±0.05, 1.14±0.04 respectively; p>0.1; Figs. 1A, 1B). Using qRT-PCR, we also measured synaptophysin mRNA levels in the wild-type and rd1 mouse retinas. Similar to our findings for the protein, the synaptophysin mRNA levels in adult rd1 mouse retina (0.84±0.08) were slightly lower but statistically similar to those in control (1.03±0.07; p>0.05; Fig. 1C).

That the levels of synaptophysin were unaltered after photoreceptor loss was surprising, because this protein is ubiquitously expressed by retinal neurons, including photoreceptors, and is therefore expected to be considerably reduced in rd1 mouse. To further confirm our finding, we measured synaptophysin levels in another animal model of retinal degeneration where photoreceptor degeneration was induced in adult wild-type mouse using N-Methyl-N-nitrosourea (MNU) [8]. We found that even MNU-induced photoreceptor loss resulted in slightly lower but statistically unaltered levels of synaptophysin in retina at various days after MNU injection (sham-injected control: 1.01±0.07, post-injection day (PID)-1: 0.99±0.09, PID-2: 0.91±0.11, PID-4: 0.92±0.05, PID-14: 0.86±0.06, PID-28: 0.99±0.08; p>0.1; Figs. 1D, 1E), except at PID-7 where synaptophysin levels (0.66±0.13) were significantly lower than in control (1.01±0.07; p = 0.044).

We reasoned that the largely unaltered levels of synaptophysin in retina even after photoreceptor loss might be because of its increased expression in the inner retinal synapses. To investigate this, we measured synaptophysin only in the IPL of adult rd1 mouse retina, using quantitative immunohistochemistry. We found that while the synaptophysin staining in the OPL of rd1 mouse disappeared almost completely, its intensity appeared to be higher in the IPL (Figs. 2A, 2B). Interestingly, a plot of staining intensity versus retinal depth through the two plexiform layers showed that the effect was greater in the Off sublamina of IPL than in the On sublamina (Fig. 2C). The levels of synaptophysin in the IPL of rd1 mouse (grayscale value; 43.4±6.8) were significantly higher than in the wild-type (21.4±3.8; p<0.05; Fig. 2D). We also measured the intensity levels separately in the On and Off sublaminas of IPL by digitally separating the outer 40% (Off) and inner 60% (On). The synaptophysin staining intensity in both On (42.1±8.1) and Off (43.5±6.2) sublaminas in rd1 mouse was approximately two-fold higher than in wild-type (23.6±4.0 and 20.4±3.9 respectively), although the increase was statistically significant in the Off sublamina (p = 0.01) but not in On sublamina (p = 0.07; Fig. 2D).

Together, these results suggested that photoreceptor loss results in upregulation of synaptic proteins in bipolar cells and/or amacrine cells. To further confirm this, we measured levels of SV2B, syntaxin-I and synapsin-I (see below).

### Ribbon-specific synaptic protein SV2B was downregulated in whole retina, but upregulated in inner retina in rd1 mouse

SV2B is expressed selectively by ribbon synapses of photoreceptors and bipolar cells [33]. Using Western blotting, we measured levels of SV2B in adult wild-type and rd1 mice. We found that the SV2B levels in rd1 mouse retina (0.46±0.09) were significantly lower than in wild-type (0.98±0.16, p<0.05; Figs. 3A, 3B). We also measured the SV2B mRNA levels in adult rd1 mouse retina. The mRNA levels in rd1 mouse (0.63±0.18) were approximately 40% lower than in wild-type (1.03±0.07) although the difference did not reach statistically significant levels (p = 0.062; Fig. 3C).

Considering the nearly total absence of photoreceptors in adult rd1 mouse, the reduction in SV2B levels was not surprising. However, this was different from our results for synaptophysin which were largely unaltered after photoreceptor loss. This difference could originate in the differential expression pattern of these proteins in inner retina – synaptophysin is expressed by bipolar cells and amacrine cells whereas SV2B is expressed only by bipolar cells. To study how bipolar cell synapses responded to photoreceptor loss, we measured SV2B levels in the IPL of rd1 mouse using quantitative immunohistochemistry.

In wild-type mouse, intense SV2B labeling was observed in the OPL while it was relatively faint in the IPL, especially in outer IPL (Fig. 3D). In rd1 mouse, OPL labeling was nearly absent whereas the labeling in the IPL was more intense and uniform (Fig. 3E). The relatively bright and large puncta in inner IPL of wild-type mouse, likely rod bipolar cell axon terminals, were smaller and fewer in the rd1 mouse, whereas the labeling in outer IPL seemed more intense in rd1 mouse (Figs. 3D, 3E). Similar to our observations for synaptophysin, the intensity profile of SV2B through retinal depth indicated that the effect was greater in the Off than in the On sublamina of IPL (Fig. 3F). The intensity of staining in the IPL of rd1 mice (grayscale value; 37.5±8.1) was more than threefold higher than in wild-type (11.6±1.5; p<0.02; Fig. 3F). Similar to synaptophysin, the SV2B staining intensity in both On (36.4±7.5) and Off (44.2±7.8) sublaminas in rd1 mouse was two- to four-fold higher than in wild-type (13.8±1.8 and 11.1±1.3 respectively), although the increase was more pronounced in the Off sublamina (p<0.01 vs. p<0.05 for On sublamina; Fig. 3G).

### Amacrine cell-specific synaptic proteins and their mRNAs were upregulated following photoreceptor loss

A comparison of results for synaptophysin and SV2B in whole retina indirectly suggested that the synaptic proteins in amacrine cells are also increased following photoreceptor loss. Considering the differential expression of synaptophysin and SV2B in inner retina, upregulation of synaptophysin in both bipolar cells and amacrine cells could have compensated for its loss in outer retina, whereas upregulation of SV2B only in bipolar cells was probably not sufficient to compensate its loss in outer retina. We investigated this directly by measuring the levels of amacrine cell-specific synaptic proteins, syntaxin-I and synapsin-I.

The levels of syntaxin-I (0.68±0.05) in rd1 mouse retina were similar to that in wild-type (0.58±0.1; p>0.1; Fig. 4A) at P-7, before the onset of photoreceptor degeneration. However, starting at P-14, the syntaxin-I levels in rd1 mouse (P-14: 0.93±0.11, P-21: 1.05±0.2, P-28: 0.99±0.13, adult: 1.47±0.17) were significantly higher than in age-matched wild-type (P-14: 0.6±0.09, P-21: 0.57±0.09, P-28: 0.63±0.09, adult: 1.02±0.11; p<0.05; Fig. 4A). This implied that the syntaxin-I upregulation occurred within a week of the onset of photoreceptor degeneration, which continued till adult stage. Similarly, MNU-induced photoreceptor loss resulted in approximately 50% increase in syntaxin-I levels on various days after MNU injection (sham-injected control: 0.65±0.07, PID-1: 0.9±0.09, PID-2: 0.94±0.06, PID-4: 0.97±0.09, PID-7: 0.9±0.08, PID-14: 0.93±0.05 and PID-28: 1.05±0.12; p<0.05 to p<0.005; Fig. 4B). Interestingly, upregulation of syntaxin-I was evident as early as one day after MNU injection, implying that the effect of photoreceptor degeneration on this protein occurred within a day of the onset of photoreceptor degeneration.

Results for synapsin-I were qualitatively similar to those for syntaxin-I, although the upregulation of synapsin-I was moderate and it took longer after photoreceptor degeneration to reach a difference of statistical significance. At P-7, synapsin-I levels in rd1 mouse (0.29±0.12), as expected, were similar to those in age-matched wild-type (0.32±0.11; p>0.1; Fig. 4C). At P-14, after the photoreceptor degeneration started, synapsin-I levels in rd1 mouse (0.89±0.17) were approximately 20% higher than in wild-type (0.75±0.09), although the difference was not statistically significant (p>0.1). However, at P-21, synapsin-I levels in rd1 mouse (1.04±0.22) were approximately twofold higher than in age-matched wild-type (0.51±0.1; p<0.05). Adult rd1 mouse also showed significantly increased levels (1.04±0.06) as compared to wild-type (0.87±0.06; p<0.05). For reasons unclear to us, the increase in P-28 rd1 mouse was not statistically significant. Synapsin-I was also upregulated in retina following MNU-induced photoreceptor degeneration (Fig. 4D). Similar to rd1 mouse, the increase in synapsin-I levels was not statistically significant in the first few days (up to PID-4) after MNU injection (p>0.1). However, from PID-7 the synapsin-I levels in MNU-injected mouse retina (PID-7: 0.88±0.05, PID-14: 0.85±0.07, PID-28: 0.89±0.11) were up to 50% higher than in sham-injected control (0.6±0.08) (p<0.05 for PID-7 and PID-14; p = 0.067 for PID-28; Fig. 4D). It is not clear why the upregulation in synapsin-I protein was milder and less consistent as compared to syntaxin-I. To understand it further, we looked at their mRNAs.

**Figure 5:**
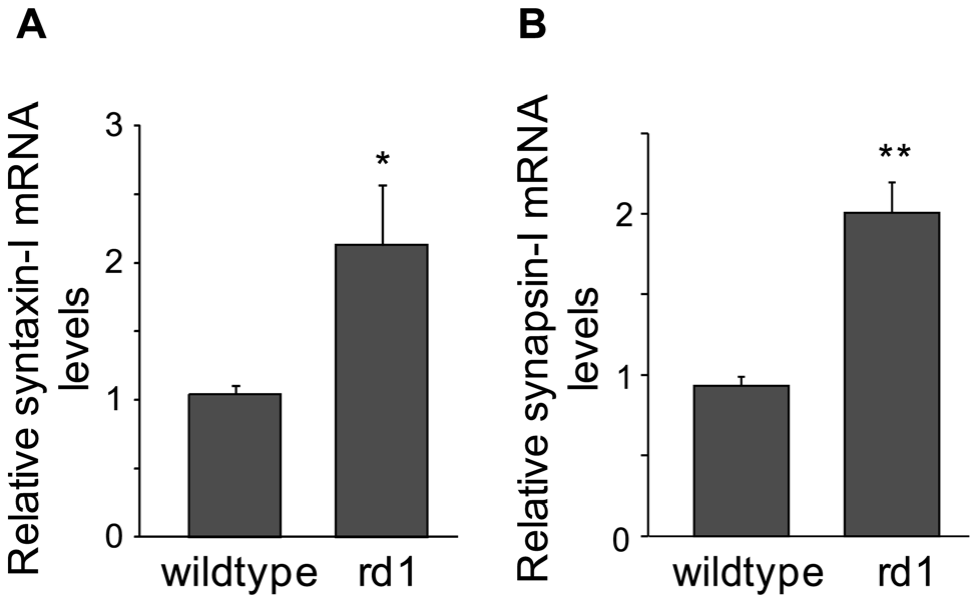
Syntaxin-I and synapsin-I mRNA levels were elevated in adult rd1 mouse retina. Relative mRNA levels (mean± SE) of both syntaxin-I (A) and synapsin-I (B) in adult rd1 mouse retina were approximately twofold higher than in wild-type (n = 6 for both). *p<0.05; **p<0.0005.

Using qRT-PCR, we also measured syntaxin-I and synapsin-I mRNA levels in adult wild-type and rd1 mouse retinas. Syntaxin-I mRNA levels in rd1 mouse (2.13±0.43) were approximately twofold higher than in wild-type (1.04±0.06; p<0.05; Fig. 5A). Similarly, synapsin-I mRNA levels in rd1 mouse (2.01±0.19) were approximately twofold higher than in wild-type (0.93±0.06; p<0.0005; Fig. 5B). It is interesting to note that unlike the changes we observed at the protein levels, the upregulation in synapsin-I mRNA was more robust than in syntaxin-I mRNA.

## Discussion

Deafferentation is known to result in structural and functional modifications in the post-synaptic neurons in the central nervous system [55]–[60]. In retina, degeneration and loss of photoreceptors leads to remodeling of the second-order retinal neurons. For example, following photoreceptor degeneration in a variety of animal models, bipolar cells and horizontal cells undergo changes, such as progressive dendritic retraction, axonal sprouting, glutamate receptor loss and redistribution, altered neurotransmitter sensitivity and ectopic synaptogenesis [1], [3]–[11], [61]–[62].

Retinal ganglion cells, the output neurons of retina are considered to be largely preserved, at least in early stages of retinal degeneration [8], [16]–[17]. However, their receptive field properties have been reported to be altered, and many of them produce aberrant spiking patterns, including spontaneous spike bursts [16], [19]. It is important to understand the mechanism underlying the altered physiological state of RGCs in retinal degeneration, because the restorative approaches, such as genetic, cellular or bionic interventions ultimately require functional integrity of these cells. Several previous reports have demonstrated that the aberrant spontaneous firing patterns in RGCs are presynaptic in origin [16], [20]–[23].

We have studied here how loss of photoreceptors affects the synaptic protein levels in bipolar cells and amacrine cells, the input neurons of RGCs. We used two animal models. The rd1 mouse is a commonly used model of inherited retinal degeneration. The MNU model was used to further confirm some of our key findings from rd1 mouse. Our central finding is that the loss of photoreceptors results in upregulation of synaptic proteins in bipolar cells and in amacrine cells. In combination with the earlier reports that both excitatory and inhibitory synaptic currents in RGCs are enhanced in rd1 mouse [16], [20]–[21], these results implied that synaptic activity in both bipolar cells and amacrine cells is increased following photoreceptor loss. This is consistent with the reports that loss of photoreceptors results in increased γ aminobutyric acid (GABA) levels and GABAa receptor sensitivity [3], [63]–[64] and larger oscillatory potentials in ERG recordings [15], and that blocking glutamate receptors abolishes the burst activity in RGCs [16], [22]. However, one caveat is that we did not study phosphorylated and non-phosphorylated synaptic proteins separately.

Synaptic proteins, including the ones examined here, have been studied in a variety of animal models of photoreceptor degeneration [8], [37]–[38]. Using a qualitative approach, these studies reported that the expression pattern of these synaptic proteins is preserved even at an advanced stage of retinal degeneration, suggesting that second and third-order retinal neurons can transmit visual information synaptically. One of these reports, although it did not quantitate, reported upregulation of synapsin in the IPL of PCD mouse [38] (see their Fig. 7C,D), which is consistent with our results for synapsin-I. Our results for synaptophysin and synapsin-I differ from an earlier report [39] which measured these proteins using bitmap image analysis and showed unaltered levels of these proteins in the IPL of young (3–4 weeks old) rd1 mouse. However, it is not clear how they calibrated the antibody dilution for these experiments. It is possible that the relatively high concentration of the antibody they used produced labeling saturation, thus obscuring the change in IPL staining intensity. We optimized the antibody concentrations for quantitative immunohistochemistry using a dose-response curve (see Experimental Procedures). Moreover, we used Western blotting to measure synapsin-I levels, which is a more sensitive method for protein quantification. The same study also reported significantly reduced levels of synaptophysin mRNA and unaltered levels of synapsin-I mRNA in the rd1 mouse retinas [39]. We found approximately 20% lower levels of synaptophysin mRNA in rd1 mouse, but the difference was not statistically significant (see Fig. 1B). One possible explanation for their not finding any increase in synapsin-I mRNA could be that they used younger animals. At least for the protein, the increase in synapsin-I levels took longer to occur in the two animal models used here (see Figs. 4C, 4D).

It is not completely clear how photoreceptor loss leads to increased synaptic activity in bipolar and amacrine cells. It is conceivable that the depletion of glutamate in the OPL following photoreceptor loss leads to activation of On bipolar cells, which could in turn increase the activity in amacrine cells. However, at least SV2B was upregulated in both On and Off sublaminas of IPL (see Fig. 3G), suggesting that both On and Off pathways are affected. In fact, the upregulation of both synaptophysin and SV2B in Off sublamina was more pronounced than in On sublamina, although the significance of this is not clear. Similarly, both On and Off RGCs are known to exhibit spike bursts, and both On and Off bipolar cells have been reported to be hyperpolarized in rd1 mouse [16], [20]. However, since these observations have been made in adult mouse, it cannot be ruled out that the On bipolar cells in rd1 mouse are more depolarized after photoreceptor loss during development and this effect is transferred passively to Off bipolar cells through glycinergic synapse. The remodeling in other components of inner retinal circuitry, such as GABA, glycine and glutamate receptors [3], [64] could possibly lead to hyperpolarization of bipolar cells in the weeks following photoreceptor loss.

The increased synaptic activity in the excitatory bipolar cells and the predominantly inhibitory amacrine cells can potentially explain how the bursts of spikes in RGCs are generated after photoreceptor loss, which are perhaps then transferred to higher visual areas in the brain [65]. The increased synaptic activity in bipolar cells would result in increased activity in GABAergic amacrine cells, which in turn would briefly inhibit the hyperactive bipolar cells through the feedback inhibition. The inhibited release from bipolar cells could then lead to reduced activity in GABAergic amacrine cells which would in turn disinhibit the bipolar cells. This self-sustaining repetitive cycle of excitation and inhibition in the bipolar cell-amacrine cell feedback loop could thus result in bursty discharge from the bipolar cells and produce spike bursts in RGCs. In fact, the GABAergic feedback inhibition of bipolar cells is known to induce temporal correlations in the quantal release from these cells, resulting in short bursts of glutamate release in normal retina [66]. It is conceivable that the increased synaptic activity (gain) in bipolar cells after loss of photoreceptors would amplify the temporal correlations in the bipolar cells and produce bursts of spikes in the postsynaptic RGC.

That the upregulation occurs soon after the onset of photoreceptor degeneration implies that it is not a result of any massive or structural remodeling. This is consistent with a recent report that pharmacologically blocking photoreceptor signaling in normal retina produced oscillatory activity in AII amacrine-On cone bipolar cell network in an acute experiment [23]. This suggests that the biochemical and the resulting physiological changes in the inner retinal neurons are possibly reversible and potentially amenable to the restorative approaches, such as retinal prostheses.

## Conclusions

We measured specific retinal synaptic proteins in two animal models of retinal degeneration to understand how inner retinal circuitry responds to photoreceptor loss. We found that levels of synaptophysin in the degenerating whole retina were largely unaltered whereas the levels of SV2B were lower. Interestingly, the levels of both these proteins were higher in the IPL of rd1 mouse retina. Since synaptophysin is expressed by bipolar cells and amacrine cells in the inner retina, its increased levels in inner retina was perhaps sufficient to compensate for its loss in outer retina, whereas the increased levels of SV2B, which is expressed only by bipolar cells in inner retina, was not. We also found that amacrine cell-specific proteins, syntaxin-I and synapsin-I were upregulated following photoreceptor loss. Increased levels of synaptic proteins in both bipolar cells and amacrine cells, combined with previous reports of increased excitatory and inhibitory synaptic currents in RGCs implies that synaptic activity in bipolar cells and amacrine cells is increased after photoreceptor loss. We propose a simplistic model to explain how increased synaptic activity in the excitatory bipolar cells and the inhibitory amacrine cells could potentially generate rhythmic spike bursts in RGCs in retinal degeneration. Interestingly, at least syntaxin-I was upregulated within a day of the onset of MNU-induced photoreceptor degeneration, suggesting that these changes are not a consequence of any massive or structural remodeling, and are therefore possibly reversible.
